# The novel drug candidate S2/IAPinh improves survival in models of pancreatic and ovarian cancer

**DOI:** 10.1038/s41598-024-56928-z

**Published:** 2024-03-16

**Authors:** Takaomi Hagi, Suwanna Vangveravong, Rony Takchi, Qingqing Gong, S. Peter Goedegebuure, Herve Tiriac, Brian A. Van Tine, Matthew A. Powell, William G. Hawkins, Dirk Spitzer

**Affiliations:** 1 Department of Surgery, Washington University School of Medicine, S. Euclid Avenue, St. Louis, MO 63110 USA; 2 Alvin J. Siteman Cancer Center, Barnes-Jewish Hospital, and Washington University School of Medicine, St. Louis, MO USA; 3 Division of Surgical Oncology, Department of Surgery, Moores Cancer Center, University of California San Diego, San Diego, CA, USA, San Diego, USA; 4 Division of Medical Oncology, Washington University School of Medicine, St. Louis, MO USA; 5 Division of Gynecologic Oncology, Department of Obstetrics and Gynecology, Washington University School of Medicine, St. Louis, MO USA

**Keywords:** Drug delivery, Drug discovery, Target validation

## Abstract

Cancer selective apoptosis remains a therapeutic challenge and off-target toxicity has limited enthusiasm for this target clinically. Sigma-2 ligands (S2) have been shown to enhance the cancer selectivity of small molecule drug candidates by improving internalization. Here, we report the synthesis of a novel drug conjugate, which was created by linking a clinically underperforming SMAC mimetic (second mitochondria-derived activator of caspases; LCL161), an inhibitor (antagonist) of inhibitor of apoptosis proteins (IAPinh) with the sigma-2 ligand SW43, resulting in the new chemical entity S2/IAPinh. Drug potency was assessed via cell viability assays across several pancreatic and ovarian cancer cell lines in comparison with the individual components (S2 and IAPinh) as well as their equimolar mixtures (S2 + IAPinh) both in vitro and in preclinical models of pancreatic and ovarian cancer. Mechanistic studies of S2/IAPinh-mediated cell death were investigated in vitro and in vivo using syngeneic and xenograft mouse models of murine pancreatic and human ovarian cancer, respectively. S2/IAPinh demonstrated markedly improved pharmacological activity in cancer cell lines and primary organoid cultures when compared to the controls. In vivo testing demonstrated a marked reduction in tumor growth rates and increased survival rates when compared to the respective control groups. The predicted mechanism of action of S2/IAPinh was confirmed through assessment of apoptosis pathways and demonstrated strong target degradation (cellular inhibitor of apoptosis proteins-1 [cIAP-1]) and activation of caspases 3 and 8. Taken together, S2/IAPinh demonstrated efficacy in models of pancreatic and ovarian cancer, two challenging malignancies in need of novel treatment concepts. Our data support an in-depth investigation into utilizing S2/IAPinh for the treatment of cancer.

## Introduction

Two subtypes of sigma receptors, sigma-1 and sigma-2, have been identified and can be distinguished by their pharmacological and functional properties^1,2^. The sigma-2 receptor TMEM97^3^, also shown to be associated with progesterone receptor membrane component-1 (PGRMC-1)^4^ is overexpressed in human malignancies, including pancreatic cancer, ovarian cancer, lung cancer, and breast cancer^5–8^. Multiple studies demonstrated that sigma-2 ligands have a high selectivity for solid tumors and can be utilized for diagnostic imaging and drug targeting purposes^9–11^. Very high doses of selective sigma-2 ligands, including SW43, can induce tumor cell death in vitro and in vivo^6,12,13^. We and others have previously shown that sigma-2 ligands are rapidly internalized into cancer cells, a highly desirable phenomenon that can be utilized to deliver therapeutic drug cargoes more efficiently into their targets, including peptides, peptidomimetics and small molecule compounds^14,15^. Taking advantage of this enhanced internalization process, we have studied a series of dual-domain drug conjugates, all of which exhibited far stronger activity profiles than either component alone or when given as isolated compounds in equimolar mixtures of the sigma-2 ligands and the various drug cargoes^16–18^.

Second mitochondria-derived activator of caspases (SMAC) is a mitochondrial protein that blocks inhibitor of apoptosis proteins (IAPs)^19^ by selectively binding to the baculovirus IAP repeat (BIR) domain of IAPs and thereby augmenting apoptosis induction^20,21^. SMAC mimetics are peptide-like synthetic small-molecule compounds with increased stability to induce apoptosis via IAP blockade^22,23^. Recently, several studies have demonstrated that SMAC mimetics induce proteasomal degradation of cellular inhibitor of apoptosis protein-1/2 (cIAP-1/2) and promote apoptosis via the extrinsic death pathway^24,25^. LCL161, a clinically explored IAP inhibitor (IAPinh), appears to be well tolerated in patients with a low toxicity profile in phase I clinical trials^26^. Although LCL161 demonstrated responses in patients with treatment-resistant myelofibrosis in a phase II clinical trial^27^, no objective responses were observed in solid tumors when tested as a monotherapy^26^.

The goal of our current study was to evaluate if the activity profile of LCL161, a clinically explored but underperforming pro-apoptotic cancer drug candidate, could be enhanced by chemical linkage to a cancer-selective sigma-2 receptor ligand (SW43). We tested the newly designed conjugate, S2/IAPinh, for its anticipated mechanistic properties relative to the parental control (LCL161). To test potential for translation, we performed a limited initial characterization of S2/IAPinh, including drug potency, mechanism and stability. We selected preclinical mouse models of pancreatic and ovarian cancer as these remain two of the most recalcitrant solid malignancies with poor prognoses^28^. Compared with other cancers, these two malignancies display a high degree of chemo-resistant disease^29,30^ and novel therapeutic strategies are urgently needed.

## Results

### Synthesis and initial characterization of S2/IAPinh

**Figure 1:**
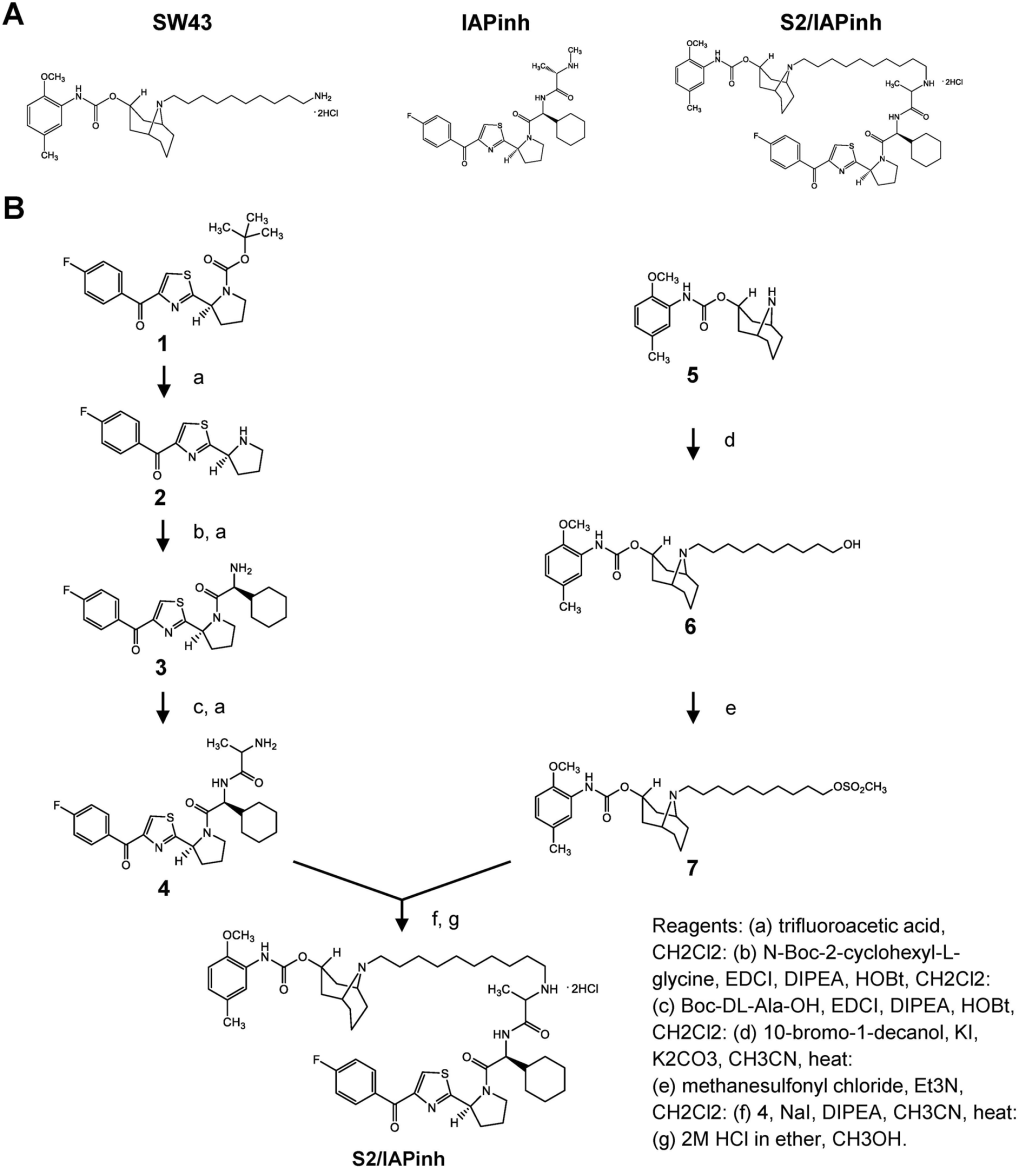
Compound structures and synthesis schematic of S2/IAPinh. (**A**) The chemical structures of SW43, IAPinh, and the chemical conjugate S2/IAPinh. (**B**) Synthesis schematic of S2/IAPinh.

**Figure 2:**
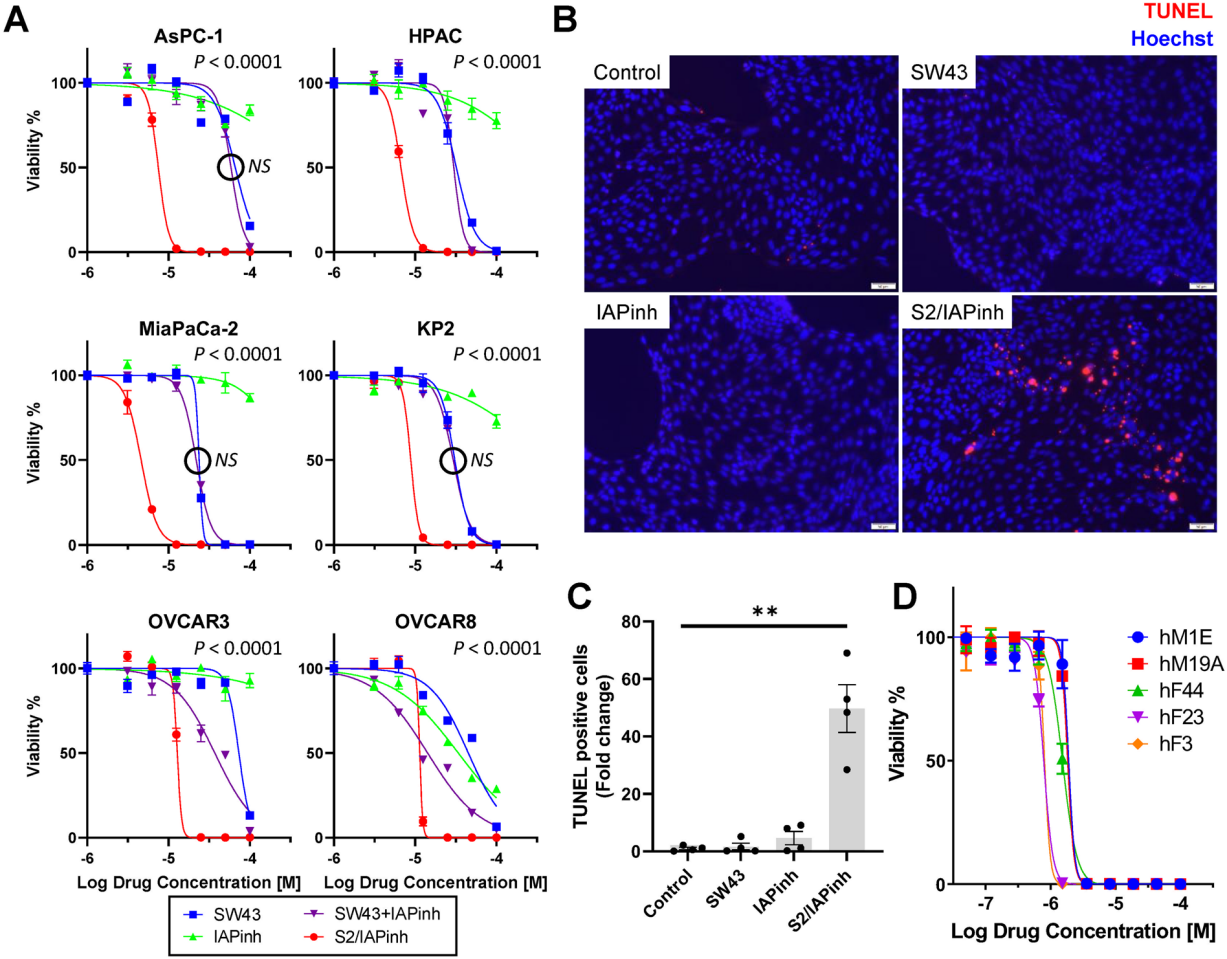
Chemical linkage to sigma-2 ligand SW43 leads to the functionally enhanced drug conjugate S2/IAPinh with increased activity over IAPinh alone. (**A**) Human pancreatic cancer cell lines AsPC-1, HPAC, and MiaPaCa-2, mouse pancreatic cancer cell lines KP2, and human ovarian cancer cell lines OVCAR3 and OVCAR 8 were treated with different concentrations (0, 3.125, 6.25, 12.5, 25, 50, and 100 µM) of SW43 alone, IAPinh alone, an equimolar mix (SW43 + IAPinh) and S2/IAPinh alone for 24 h and cell viability was determined by CellTiter-Glo Luminescent Viability Assay (Promega). Two-way ANOVA; NS = not significant. (**B**) Representative images of TUNEL labeled apoptosis cells in HPAC cells treated with vehicle, SW43 (6 µM), IAPinh (6 µM), or S2/IAPinh (6 µM) for 6 h. Nuclei were stained in blue with Hoechst, TUNEL positive cells are in red. Scale bars are equal to 50 µm. (**C**) Quantification of TUNEL positive cells per area in each treatment group. Data are shown as means ± SEM; **P < 0.01. (**D**) Organoid cultures from patients with pancreatic cancer (hF3, hF23, hF44, hM1E, hM19A) were treated with escalating concentrations of S2/IAPinh (0, 0.01, 0.02, 0.05, 0.12, 0.28, 0.66, 1.52, 3.6, 8.2, 18.76, 43.2, and 100 µM) for 5 days and cell viability was determined by CellTiter-Glo Luminescent Viability Assay (Promega) (n = 4 per cell line).

S2/IAPinh was synthesized by chemically linking the sigma-2 ligand SW43 with IAPinh (LCL161) (Fig. 1A). Briefly, the commercially available compound 1 was deprotected and coupled with substituted amino acids to result in compound 4. N-Alkylation of compound 4 and 7 (prepared from compound 5) resulted in the final product, S2/IAPinh (Fig. 1B and Ref.^31^). Cell viability assays were performed using six pancreatic and ovarian cancer cell lines in vitro to demonstrate the efficacy of the new drug candidate relative to the parental compounds. More specifically, treatment conditions included the individual components in isolation (IAPinh alone and sigma-2 ligand alone [SW43]), equimolar mixtures of the isolated components of the conjugate (SW43 + IAPinh) and the conjugate alone (S2/IAPinh). We found that SW43 alone showed a mild degree of cytotoxicity across all cell lines with IC_50_ values ranging from 23.8 to 75.2 µM (Fig. 2A and suppl. Table S1). IAPinh alone, as high as 100 µM, did not show significant cytotoxic effects in the majority of solid tumor cell lines, with the exception of the ovarian cancer cell line OVCAR8, which was significantly more sensitive to IAPinh compared to all other cell lines tested (IC_50_ of 34.5 µM, Fig. 2A). The treatment effects of equimolar mixtures of SW43 and IAPinh were dominated by the intrinsic cytotoxicity of the sigma-2 ligand SW43 (IC_50_ range from 22.0 to 58.8 µM, Fig. 2A). In contrast, S2/IAPinh showed the enhanced cytotoxicity compared to the controls in all cancer cell lines with IC_50_ values ranging from 4.6 to 12.8 µM (Fig. 2A). These data demonstrate that the chemical linkage between IAPinh (LCL161) and SW43, was feasible and substantially improved the activity profile of LCL161 in vitro.

Apoptotic cell death is the anticipated mode of action of IAPinh and has been confirmed for LCL161^32^. We postulated that our novel drug conjugate would retain these key characteristics and performed selected confirmatory studies, including a terminal deoxynucleotidyl transferase (TdT) dUTP Nick-End Labeling (TUNEL) assay. This assay detects cells undergoing extensive DNA fragmentation during the late stages of apoptosis. In order to demonstrate broad applicability and ensure reproducibility, we selected two cancer types including a human pancreatic cancer cell line (HPAC) and a human ovarian cancer cell line (OVCAR8) for these assays. At the doses used in our study, only the S2/IAPinh treated cells showed significant generation of DNA breaks (TUNEL positivity), while all other treatment groups, including SW43 alone, IAPinh alone, and the non-treated control cells did not (Fig. 2B,C, and suppl. Fig. S1). These data suggest that an apoptotic cancer cell death was induced by S2/IAPinh.

The potential clinical relevance of our drug development efforts was tested in primary organoid cultures derived from pancreatic cancer patients (suppl. Table S2A). We found that all five PDAC organoids, including those known to be resistant to other treatment regimens, were highly sensitive to the conjugate with IC_50_ values ranging from 0.8 to 1.9 µM (Fig. 2D and suppl. Table S2B).

**Figure 3:**
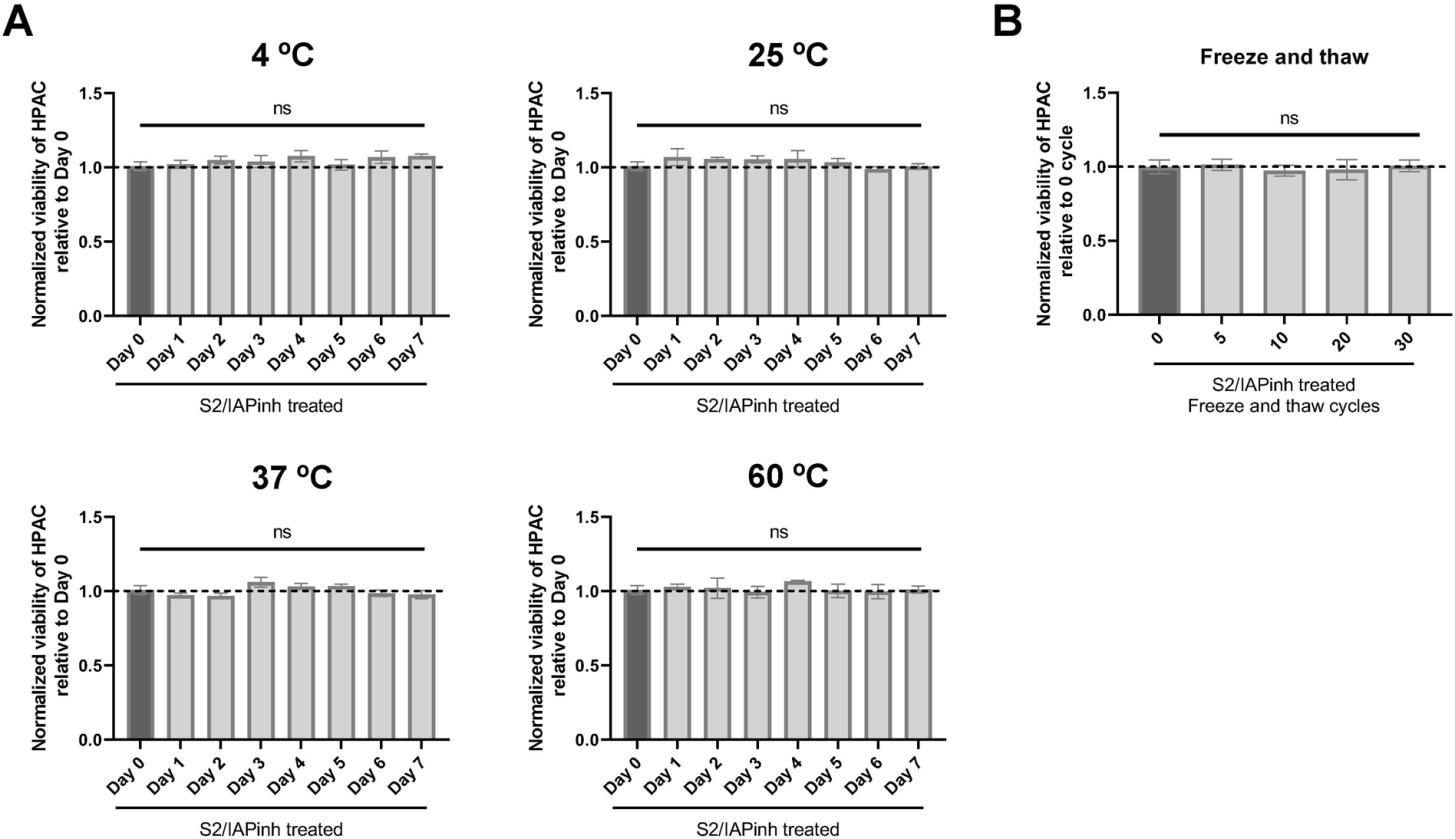
Physicochemical stability evaluations of S2/IAPinh. (**A**) Killing activity of S2/IAPinh (6 µM stock solution) stored at 4 °C, 25 °C, 37 °C, or 60 °C using HPAC reporter cells. An aliquot of the stock solution was frozen immediately (Day 0; black bar, baseline activity). Additional aliquots at the respective temperature condition were removed in 24 h intervals and immediately frozen. At the conclusion of the 7-day experiment, all samples were thawed and subjected to a viability assay on HPAC cells. The day 0 aliquot served as a reference (black bars) with a baseline killing capacity of 77.7% (normalized as dotted lines). (**B**) Killing activity of S2/IAPinh (6 µM stock solution) following a series of freeze/thaw cycles. An aliquot of the stock solution was frozen immediately (0 freeze/thaw cycles, black bar, baseline activity). Additional aliquots were removed following 5, 10, 20 and 30 freeze/thaw cycles and stored on ice prior to functional evaluation. At the conclusion of the cycling experiment, all samples were thawed and subjected to a viability assay on HPAC cells. The non-cycled aliquot served as a reference (black bar) with a baseline killing capacity of 77.7% (normalized as dotted line).

A key requirement of a clinically viable (cancer) drug candidate relates to its physicochemical properties, including its stability under standard storage and handling conditions. As a preliminary foray into clinical viability, we tested the stability of S2/IAPinh in solution under storage and clinical conditions including temperature ranges, and freeze/thaw cycling. As a first readout, we perormed cell viability determinations employing the pancreatic cancer cell line HPAC as a reporter and a fixed drug concentration that induced 70% cell death, a sublethal concentration in the linear, non-plateau range that allowed for the identification of losses in drug activity, potentially caused during the planned storage/handling conditions. S2/IAPinh did not appear to lose its efficacy after being stored for seven days at 4 °C, 25 °C and 37 °C physiologic body temperature. In order to further increase the stringency of stability testing, we discovered that S2/IAPinh did not even lose functional activity after storage at non-physiologic 60 °C (Fig. 3A). Furthermore, the conjugate activity remained unaffected even after submitting S2/IAPinh up to 30 freeze/thaw cycles (Fig. 3B). These data suggest that S2/IAPinh was chemically stable under clinically relevant storage/handling conditions. While further and more in-depth drug characterizations might be necessary in the future, a failure at this early stage to meet basic stability requirements would have potentially precluded further development of S2/IAPinh as a viable drug candidate.

### S2/IAPinh causes cancer cell death through induction of apoptosis

**Figure 4:**
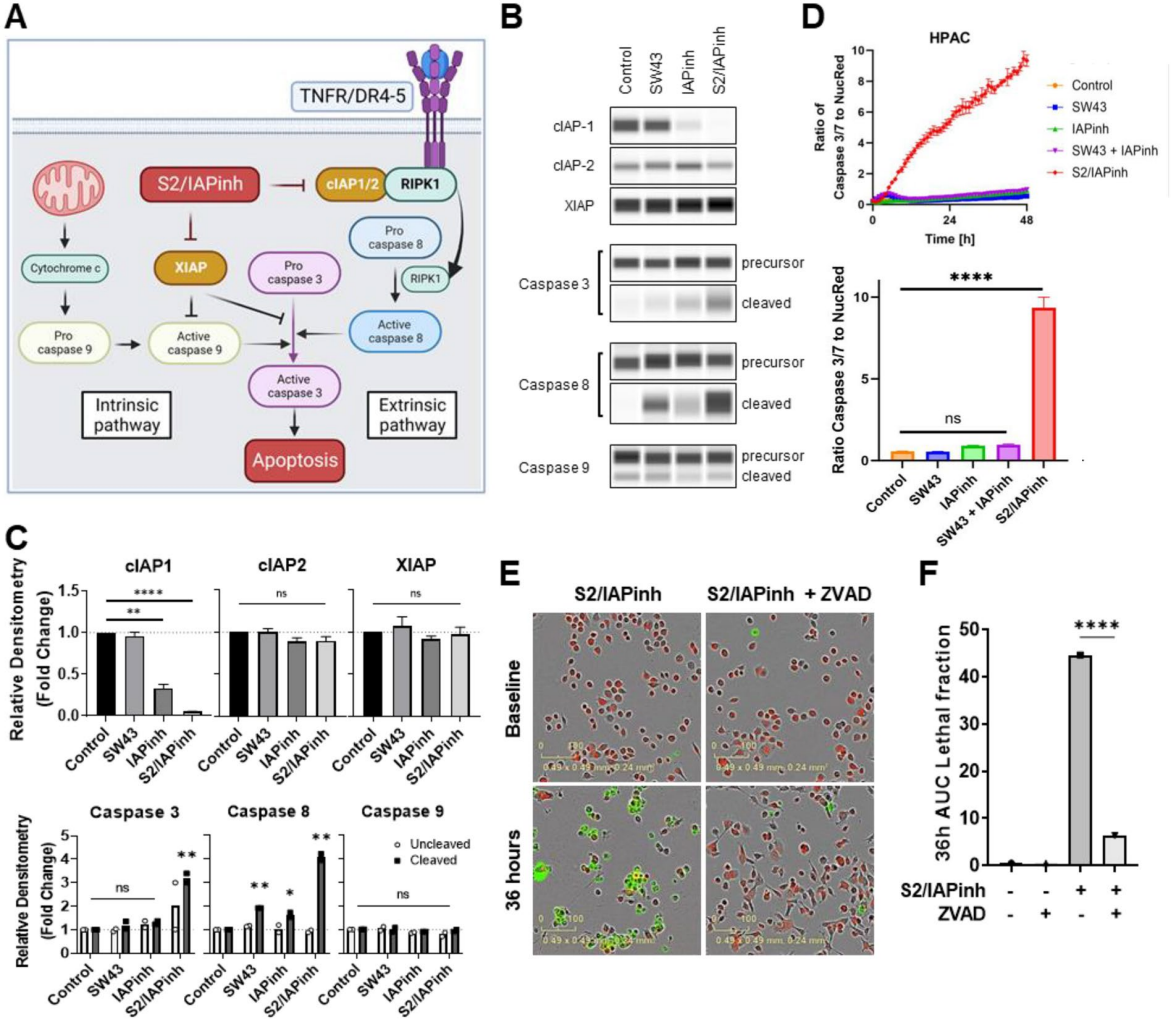
S2/IAPinh induces activation of the extrinsic apoptosis pathway via degradation of cIAP-1. (**A**) Schematic diagram of the intrinsic and extrinsic apoptosis pathways: arrows represent stimulation. TNFR, tumor necrosis factor receptor; DR4-5, death receptor 4–5; cIAP-1/2, cellular inhibitor of apoptosis protein-1/2; RIPK1, Receptor-interacting serine/threonine-protein kinase 1. (**B**) Protein expression of cIAP-1, cIAP-2, and XIAP in HPAC cells treated with vehicle, SW43 (10 µM), IAPinh (10 µM), or S2/IAPinh (10 µM) for 6 h. The precursor and cleaved forms of caspases 3, 8, and 9 were also analyzed for these cells using Wes automated capillary blotting system (Protein Simple). (**C**) Quantification of protein expression. Relative densitometry of each band normalized to the total protein. Data shown as means ± SEM. **P < 0.01, **** P < 0.0001. (**D**) Ratio of Caspase 3/7 counts to NucRed counts in HPAC cells treated with vehicle, SW43 (10 µM), IAPinh (10 µM), combination of SW43 (10 µM) and IAPinh (10 µM), or S2/IAPinh (10 µM) measured by the IncuCyte system (Sartorius). Bar graph shows the ratio of Caspase 3/7 at 48 h for each treatment. Data shown as means ± SEM. ****P < 0.001. (**E**) Activity of cell death in AsPC-1 cells was measured using YOYO-1 iodide on the IncuCyte (Sartorius). Representative images of AsPC-1 cells treated with or without Z-VAD-FMK and S2/IAPinh (10 uM) at baseline and 36 h after treatment. Scale bars are equal to 20 µm. (**F**) The AUC of lethal fraction at 36 h. Data shown as means ± SEM. ****P < 0.0001.

To further confirm the expected mechanism of action, we explored the previously reported death-inducing activities of IAPinh, including the proteolytic degradation of cIAPs. We tested the effects of S2/IAPinh on cIAP-1/2 protein levels in vitro (Fig. 4A). Similar to IAPinh alone, exposure to S2/IAPinh correlated with a significant decrease in cIAP-1 protein levels, while cIAP-2 appeared to be unaffected in both pancreatic (HPAC, Fig. 4B and C) and ovarian cancer cells (OVCAR8, suppl. Fig. S2A and B). Similarly, no changes in XIAP abundancy were identified, not unexpected, as SMAC-mediated apoptosis induction occurs without proteolytic XIAP degradation (Fig. 4B,C, suppl. Fig. S2A, and B). Moreover, activation of caspases 3 and 8 was induced by S2/IAPinh treatment in both HPAC (*P* = 0.008 and *P* = 0.002) and OVCAR8 cells (*P* = 0.014 and *P* = 0.022), while activated caspase 9 levels appeared to be unaffected by S2/IAPinh (Fig. 4B,C, suppl. Fig. S2A, and B).

Since IAPinh appeared to be as effective as S2/IAPinh in reducing cIAP-1 levels over a 6 h time period (Western blotting) but failed to exert a comparably strong cell death program (cell viability assay), we investigated the temporal aspects of drug activity for both compounds. We evaluated the progression of caspase 3/7 activity over time induced by SW43 alone, IAPinh alone, equimolar mixtures (SW43 + IAPinh) and S2/IAPinh alone. The pancreatic cancer cell line HPAC and the ovarian cancer cell line OVCAR8 were treated for 48 h and monitored continuously using fluorescent probe activation as detection system of activated (cleaved) caspase 3/7. In HPAC cells, treatment with S2/IAPinh significantly increased the onset and the overall activity of caspase 3/7 relative to all other treatments, while none of the other conditions (alone and as equimolar mixtures) resulted in significant caspase activation (*P* < 0.001, Fig. 4D). Similarly, OVCAR8 cells treated with S2/IAPinh significantly increased the overall caspase 3/7 activity in comparison to any other treatment condition (*P* < 0.001, suppl. Fig. S2C). Of note, while caspase 3/7 activation was more pronounced in IAPinh treated OVCAR8 cells (*P* < 0.05, suppl. Fig. S2C), its combination with SW43 (IAPinh + SW43) did not further increase caspase 3/7 activity and was dominated by the IAPinh component of the mixture (ns, suppl. Fig. S2C), in analogy to the lack of SW43 contribution to caspase 3/7 activation in HPAC cells described above (Fig. 4D).

In order to confirm the involvement of caspases in the mechanism of cell death execution of S2/IAPinh, the pan-caspase inhibitor Z-VAD-FMK was used to block this effector arm of apoptosis. Both, AsPC-1 and OVCAR8 cells were strongly protected from apoptosis in the presence of Z-VAD-FMK. This result is indicative of the importance of caspase activation in S2/IAPinh-mediated cell death (Fig. 4E,F, suppl. Fig. S2D, and E).

Overall, these data demonstrate that the superior activity profile of S2/IAPinh is likely mediated through a more rapid and/or efficient cellular uptake mechanism into the target cells. The mechanism of cell death with S2/IAPinh resembles that of the cargo component, IAPinh, is demonstrated by efficient cIAP-1 degradation, leads to strong caspase 3 and caspase 8 activation, which can be reversed via global caspase inhibition with Z-VAD-FMK, leading to protection against cell death.

### S2/IAPinh exhibits antitumor activity in vivo

Prior to exploring S2/IAPinh in an in vivo model of treatment efficacy, we assessed its toxicity profile in C57BL/6 wild type male and female mice at various drug doses. There were no significant differences in complete blood counts (CBC) and serum chemistries (blood urea nitrogen, creatinine, total protein, aspartate transferase, and alanine transaminase) between the vehicle group and the S2/IAPinh treated group (suppl. Fig. S3A and B) across the ranges tested. Necropsy and histopathological examination of major organs including liver, kidney, lung, and brain revealed no evidence of overt toxicities except for mild to moderate inflammation in the peritoneal cavity close to the drug injection site (suppl. Fig. S3C).

**Figure 5:**
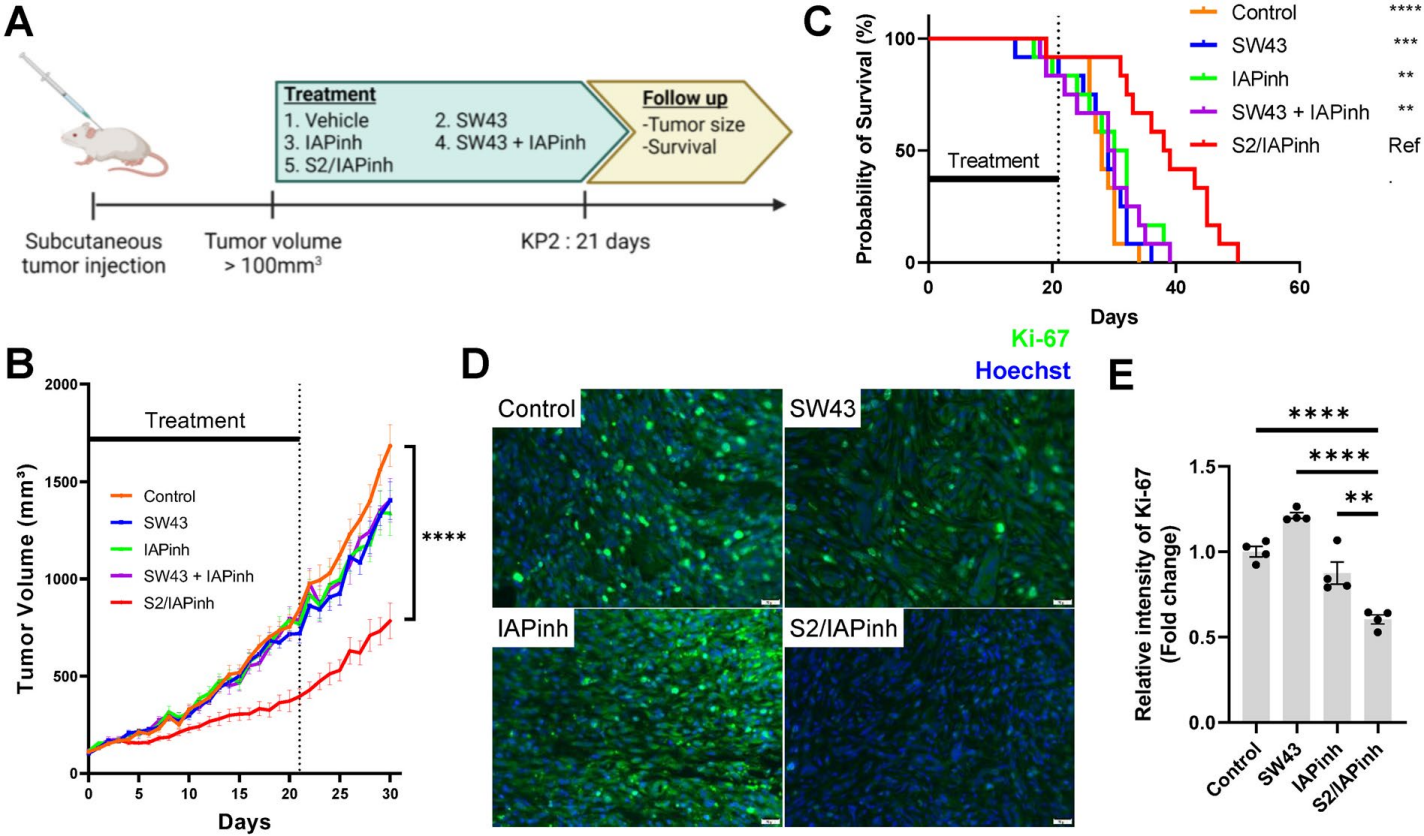
Antitumoral effect of S2/IAPinh in murine subcutaneous tumor models. (**A**) KP2 xenograft subcutaneous tumor model using 6-week-old female C57BL/6 mice (12 mice per group) were start treated when the tumor reached approximately 100 mm^3^. Drug were administered by intraperitoneal injection every day for 21 days with either vehicle (25% cremophor in H_2_O), 25 µmols/kg/day of SW43 alone, 25 µmols/kg/day of IAPinh alone, combination of 25 µmols/kg/day of SW43 and 25 µmols/kg/day of IAPinh, or 25 µmols/kg/day of S2/IAPinh. (**B**) Tumor volume of mice during or after each treatment (n = 12/group). There were no significant differences between vehicle, SW43, IAPinh, and combination of SW43 and IAPinh. Data shown as means ± SEM. **** P < 0.0001. (**C**) Kaplan–Meier survival curve of the mice in each treatment group is shown (n = 12/group). There were no significant differences in survival between vehicle, SW43, IAPinh, and combination of SW43 and IAPinh treated group. **P < 0.01, *** P < 0.001, **** P < 0.0001. (**D**) Ki-67 staining for tumor samples collected 48 h after each treatment (n = 4/group). Representative images of Ki-67 staining. Nuclei were stained in blue with Hoechst, Ki-67 in green. Scale bars are equal to 20 µm. (**E**) Quantification of Ki-67 staining per area in each treatment group 48 h after each treatment (n = 4/group). Data are shown as means ± SEM; **P < 0.01, **** P < 0.0001.

Preclinical efficacy testing of S2/IAPinh was performed in a syngeneic model of murine pancreatic cancer and in a xenograft setting of human ovarian cancer using subcutaneous flank tumors. In the KP2 syngeneic PDAC model, C57BL/6 wild type mice were used as hosts (Fig. 5A). Compared to the vehicle control group, SW43, IAPinh, and an equimolar mix of SW43 and IAPinh showed no significant differences in tumor growth delay at 30 days after treatment start (*P* = 0.56, *P* = 0.67, and *P* = 0.67, respectively; Fig. 5B). In contrast, S2/IAPinh treated animals responded with a significant delay in tumor growth rate compared to the vehicle control, SW43 alone, IAPinh alone, and the SW43 + IAPinh combination (*P* < 0.001, *P* = 0.003,* P* = 0.002, and *P* = 0.002, respectively). The differential tumor growth curves were reflected in the median survival of the animals treated with the conjugate S2/IAPinh (39 days, range 19–50 days), significantly longer in comparison to all other treatment groups, including vehicle (median 28 days, range 18–34 days; *P* < 0.001), SW43 alone (median 29 days, range 14–36 days; *P* < 0.001), IAPinh alone (median 31 days, range 17–39 days; *P* = 0.005), and the SW43 + IAPinh combination (median 29.5 days, range 18–39 days; *P* = 0.002) (Fig. 5C).

Similar effects were demonstrated in a subcutaneous xenograft model of ovarian cancer using OVCAR8 cells and athymic nude mice. Here, the tumor size in S2/IAPinh treated mice at day 50 was significantly smaller than those of vehicle control (*P* = 0.008), SW43 alone (*P* < 0.001), IAPinh alone (*P* < 0.001), and also the equimolar combination of SW43 + IAPinh (*P* = 0.001) (suppl. Fig. S4A). Extension of animal survival was only noted in mice treated with S2/IAPinh (median 60.5 days, range 34–76 days) compared with all other treatment groups, i.e. vehicle control (median 45.5 days, range 28–64 days; *P* = 0.008), SW43 alone (median 39 days, range 27–63 days; *P* = 0.002), IAPinh alone (median 47 days, range 29–67 days; *P* = 0.007), and the SW43 + IAPinh combination (median 49 days, range 25–63 days; *P* = 0.009) (suppl. Fig. S4B). Throughout the course of the efficacy experiments, both C57BL/6 and athymic nude mice appeared well regardless of treatment regimen. No treatment group lost significant body weight (as a measure of putative drug toxicities) and we observed no deaths related to drug therapy (suppl. Fig. S4C and S5A).

As a gauge for in vivo activity of drug treatment at the tumor site, we assessed the cell proliferation marker Ki-67 via immunofluorescent histochemistry 48 h after drug injection. S2/IAPinh treated animals and demonstrated a significantly lower proliferation index compared to all other treatment groups, including vehicle control (*P* < 0.001), SW43 alone (*P* < 0.001) and IAPinh alone (*P* < 0.001) (Fig. 5D,E and suppl. Fig. S5B). These data are consistent with the observed delay in tumor progression found only in S2/IAPinh treated mice.

**Figure 6:**
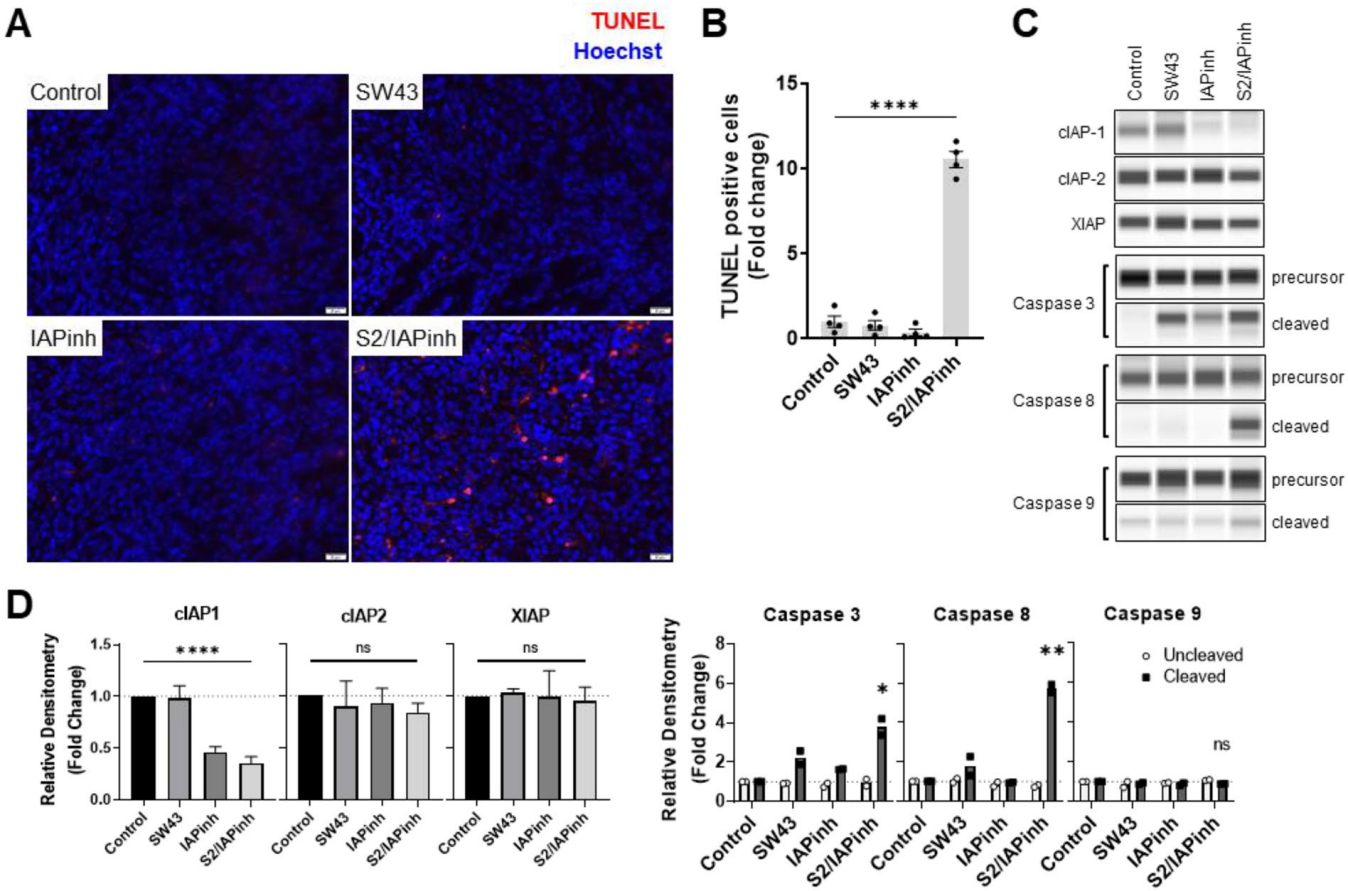
S2/IAPinh induces tumor cell death via activation of the extrinsic apoptosis pathway in vivo. KP2 tumor samples collected from animals at 48 h after the treatment with either vehicle, SW43, IAPinh, or S2/IAPinh, were used for analyses. (**A**) Representative images of TUNEL labeled apoptosis cells in KP2 tumor samples of each group. Nuclei were stained in blue with Hoechst, TUNEL positive cells are in red. Scale bars are equal to 20 µm. (**B**) Quantification of TUNEL positive cells per area in each group. Data are shown as means ± SEM; ****P < 0.0001. (**C**) Protein expression of cIAP-1, cIAP-2, and XIAP in KP2 tumor samples of each group. The precursor and cleaved forms of caspases 3, 8, and 9 were also analyzed for these cells using Wes automated capillary blotting system (Protein Simple). (**D**) Quantification of protein expression. Relative densitometry of each band normalized to the total protein. Data shown as means ± SEM. *P < 0.05, **P < 0.01, **** P < 0.0001.

As a means to confirm in vivo pathway activation of S2/IAPinh, KP2 tumors were surgically removed 48 h after drug treatment and prepared for TUNEL analysis to detect late apoptosis-induced DNA double strand breaks. Our data revealed a pronounced number of TUNEL-positive tumor cells in S2/IAPinh treated animals, while all other treatment groups lacked a marked induction of DNA damage, including the vehicle control, SW43 alone and IAPinh alone (*P* < 0.001) (Fig. 6A and B).

Western blot demonstrated degradation of cIAP proteins consistent with IAPinh-mediated drug activity. Using the same murine KP2 PDAC tumor model, we observed that IAPinh and S2/IAPinh treated tumors showed significant decreases in cIAP-1 protein levels when compared with controls and are in accordance with the results of our in vitro drug characterization efforts described above (Fig. 6C and D). Also, and consistent with our in vitro results, IAPinh and S2/IAPinh treatment had no impact on intratumoral cIAP-2 and XIAP protein levels. And finally, activation of caspases 3 and 8 were significantly upregulated in S2/IAPinh treated tumors (*P* = 0.022 and *P* = 0.002), while caspase 9 activity remained unaltered in all treatment groups.

## Discussion

The sigma-2 receptor is over-expressed in tumor cells and the corresponding ligands can be utilized as cytotoxic agents in experimental cancer models at high doses^15,33^. We have previously demonstrated that conjugation of small molecules to sigma-2 ligands enables high-efficient cargo delivery, while the mechanistic properties of the payloads are preserved^16,17,34^. SMAC mimetics represent promising candidate drugs as they interact robustly with the family of IAP proteins, which are often highly expressed in numerous human malignancies^35–37^. Upregulation of these pro-survival mechanisms are partly to blame for the limited success of cancer therapies^38–40^. IAPinh (LCL161), a clinically explored SMAC mimetic, has been tested in several cancer types but failed to exert clinical benefit for the patients^41–43^. By chemically linking IAPinh to the sigma-2 ligand SW43, we aimed to increase the overall potency of a biologically sound approach and create a novel cancer drug candidate (S2/IAPinh). Given the perceived clinical need, we have begun characterizing S2/IAPinh in vitro and in preclinical models of pancreatic and ovarian cancer.

We were able to demonstrate that S2/IAPinh enhanced cell death in pancreatic and ovarian cancer cell lines, especially in comparison to the parental control (LCL161/IAPinh alone). These results were corroborated in vivo using syngeneic (KP2 murine PDAC) and xenograft mouse models of cancer (OVCAR8 human ovarian cancer), without evidence of overt systemic toxicities.

Of particular interest from a translational drug development perspective was the finding that S2/IAPinh was quite effective when assessed in vitro utilizing organoid cultures derived directly from pancreatic cancer patients (Fig. 2D), known to be resistant to standard chemotherapies, including gemcitabine, paclitaxel, and oxaliplatin^44^. As such, our data suggest that S2/IAPinh could be a viable second line treatment option in the fight against chemotherapy-resistant pancreatic cancers.

Mechanistically, cIAP-1 protein was efficiently degraded by both, IAPinh and S2/IAPinh, indicative of a retained mode-of-action of the IAPinh cargo component of the conjugate, a critical and desired feature of the sigma-2 ligand-based drug delivery technology. Along these lines, and according to the established activity profile of IAPinh (LCL161), it was not surprising that both compounds did not affect the protein levels of cIAP-2 and XIAP in HPAC, OVCAR8, and KP2 cells. This drug characteristic differs slightly from our previously designed conjugate SW IV-134, in which the sigma-2 ligand SW43 was chemically linked to the SMAC mimetic SW IV-52 (cargo), a XIAP inhibitor with a strong potency to degrade cIAP-1 and cIAP-2 (Refs.^16,17,45^).

In order to formally interrogate the mechanistic subtleties between S2/IAPinh and SW IV-134, we performed experiments with SW IV-134 and assessed its impact on cIAP-1/2 degradation and on XIAP levels in HPAC and OVCAR8 cells. Here, SW IV-134 caused a significant reduction of cIAP-1 and cIAP-2 protein levels, while XIAP levels remained unchanged (data not shown). Both outcomes are consistent with our previous study and the reported mode of action of SW IV-52. Considering that S2/IAPinh and SW IV-134 only differ with regard to affecting cIAP-2 protein levels in pancreatic (HPAC) and ovarian cancer cell lines (OVCAR8), we conclude that the overall mechanistic properties of each drug conjugate, in the context of a given tumor type, are consistent with the activity profile of its related cargo, i.e. cIAP-1/2 degradation with SW IV-134 via its cargo (SW IV-52, SMAC mimetic) but only cIAP-1 degradation with S2/IAPinh via its cargo (LCL161, IAPinh).

Further mechanistic evaluation of S2/IAPinh identified strong activation of caspases 3 and 8 but little changes in the activation pattern of caspase 9. According to a number of mechanistic studies on the impact of cIAP-1/2 signaling, it is well established that its degradation is intimately associated with ubiquitin-mediated activation of tumor necrosis factor receptor-associated factor 2 (TRAF2) and receptor-interacting protein 1 (RIP1). Through this mechanism, tumor necrosis factor receptor 1 (TNF-R1) mediated caspase 8 activation triggers the extrinsic pathway of apoptosis^46,47^, while XIAP represents a key antagonist of both forms of apoptosis^21,48,49^. Given that S2/IAPinh only affects cIAP-1 protein levels, we propose that S2/IAPinh induced cell death is strongly related to the extrinsic pathway of apoptosis but has only limited activity on the intrinsic pathway of programmed cell death.

Our study on the characterization of the novel drug candidate S2/IAPinh identified a strong activation pattern of caspase 3 compared to the same doses of the isolated components of the conjugate, SW43 and IAPinh, in vitro. These results were replicated in vivo, where S2/IAPinh treatment showed significant impact on tumor growth retardation and animal survival, whereas treatment with SW43 and IAPinh alone did not confer any treatment benefits. Even though degradation of cIAP-1 was triggered to a similar degree by both IAPinh and S2/IAPinh in vivo when administered at equimolar concentrations, the IAPinh treated group did not show any signs of treatment benefit. This result is reminiscent of the lack of LCL161 therapy responses in cancer patients^26^ and similar to the results we have seen in our in vitro investigations relating to the tumor cell killing capacities of the compounds. To this point, we can only speculate about the exact cause(s) of this apparent discrepancy between cIAP-1 degradation ability and treatment efficacy regarding IAPinh and S2/IAPinh. We previously reported that multiple pathways can be induced by the sigma-2 ligands themselves and that these effects can vary between different cell lines and tumor types^33,50^. Therefore, we hypothesize that the additional mechanistic activity of the sigma-2 ligand component of our drug conjugate may contribute in some way to the overall potency of S2/IAPinh, a scenario that is currently under investigation in our laboratory.

Both the pancreatic and ovarian cancer models demonstrated a treatment response to S2/IAPinh as demonstrated by a delay in tumor size and an extension of animal survival. However, tumor eradication was not achieved with monotherapy. Future studies will thus be necessary to further improve the therapeutic window of S2/IAPinh in combination with suitable partner dugs, a concept that has been recently successfully employed in the context of a PDX model in ovarian cancer, where the drug conjugate, SW IV-134 (a potent XIAP inhibitor with strong degradation capacity for cIAP1/2, see above), was used in combination with cisplatin, which led to a complete tumor eradication^51^.

In summary, we have developed a novel sigma-2 ligand-based drug conjugate coined S2/IAPinh and demonstrated its efficacy in two of the most recalcitrant human malignancies—pancreatic and ovarian cancer. This new drug conjugate induces a rapid degradation of its intended target, cIAP-1, while it also leads to activation of caspase 8, a key component of the extrinsic pathway of apoptosis. The mechanistic findings reported here suggest that this new drug conjugate deserves investigation as an additional treatment option for cancer therapy. While it might work as a single agent it’s mechanism and the targeted nature of the compound suggest the optimal approach might be as a combination with suitable partner drugs that activate complementary signaling cascades on the path toward high efficient cancer therapy.

## Materials and methods

The primary goal of our current study was to characterize a novel chemical conjugate between the sigma-2 ligand SW43 (S2) and the IAP inhibitor LCL161 (IAPinh), resulting in S2/IAPinh. It’s synthesis scheme as well as its detailed biochemical and functional characteristics in vitro and its applicability as a putative new drug candidate in preclinical mouse models of pancreatic and ovarian cancer are described.

### Compounds

The chemical structures of sigma-2 ligand SW43, IAPinh (LCL161), and the chemical conjugate S2/IAPinh is shown in Fig. 1A. The synthesis of S2/IAPinh is outlined in Fig. 1B.

### Cell lines

The human pancreatic cancer cell lines AsPC-1, HPAC, and MiaPaCa-2, and the human ovarian cancer cell line OVCAR3 was obtained from the American Type Culture Collection (ATCC, Manassas, VA). The OVCAR8 cell line was a generous gift from Dr. Katherine C. Fuh^52^. The mouse KP2 line was derived from pancreatic ductal adenocarcinoma (PDAC) tissue obtained from p48-CRE/LSL-Kras^G12D^/p53^flox/+^ mice (backcrossed C57BL/6, n = 6) as previously described^18^. AsPC-1, OVCAR3, and OVCAR8 cells were cultured in RPMI Medium 1640 (Life Technologies, Grand Island, NY) with 10% FBS and Penicillin–Streptomycin (10,000 U/mL) (Life Technologies, Grand Island, NY), HPAC, MiaPaCa-2, and KP2 cells were cultured in 45% Dulbecco’s Modified Eagle Medium (DMEM) (Life Technologies, Grand Island, NY) and 45% Ham’s F-12 Nutrient Mix (Life Technologies, Grand Island, NY) with 10% FBS and Penicillin–Streptomycin (10,000 U/mL) (Life Technologies, Grand Island, NY).

For transfection of Nuclight Red cell lines, all cell lines were transduced with IncuCyte NucLight Red lentivirus reagent (EF-1 Alpha promoter, Puromycin selection) (4625, Sartorius, Ann Arbor, MI). Briefly, cells were seeded and allowed to adhere for 24 h to reach approximately 20–40% confluence. NucLight reagent was diluted in either RPMI Medium or DMEM containing 8 μg/mL polybrene (TR1003G, Fisher Scientific, Pittsburgh, PA) and added to cells for 48 h. Media was then replaced with fresh medium containing 3 μg/mL puromycin (ant-pr-1, InvivoGen, San Diego, CA). Red fluorescence was monitored on the IncuCyte ZOOM system (Sartorius, Ann Arbor, MI), and analyzed using the IncuCyte image analysis software (Sartorius, Ann Arbor, MI). All cell lines were maintained in a humidified incubator at 37 °C with 5% CO_2_, were tested for mycoplasma, and were cultured for less than 3 months post thawing.

### Human tissues

Normal pancreatic tissues were obtained from the islet transplant program at the University of Miami Miller School of Medicine as previously described (Ref.^53^). Briefly, pancreatic cancer tissue was obtained from patients undergoing surgical resection or tissue biopsy at Memorial Sloan Kettering, Stony Brook University (GI Cancer Clinical Resource Core), Johns Hopkins University, Northwell Health, Weill Cornell University, University of California, Davis, Thomas Jefferson University Hospital, MD Anderson Cancer Center, Washington University St. Louis, and St. Francis Hospital. Autopsy specimens from metastatic sites were obtained from the Rapid Autopsy Program at University of Nebraska Medical Center and Washington University St. Louis. All tissue donations and experiments were reviewed and approved by the Institutional Review Board of Cold Spring Harbor Laboratory and all clinical institutions. Written informed consent was obtained prior to acquisition of tissue from all patients. The studies were conducted in accordance with recognized ethical guidelines (Declaration of Helsinki). Samples were confirmed to be tumor or normal based on pathologist assessment.

### Human organoids

Human organoids were prepared as previously described (Ref.^44^). Briefly, tissues were minced and incubated in digestion media (1 mg/mL Collagenase XI, 10 µg/mL DNAse I, 10.5 µmol/L Y-27632 in Human Complete Medium) at 37 °C with mild agitation for up to 1 h. Cells were plated with Matrigel and grown in Human Complete Feeding Medium: advanced DMEM/F12, HEPES 10 mmol/L, Glutamax 1 × , A83-01 500 nmol/L, hEGF 50 ng/mL, mNoggin 100 ng/mL, hFGF10 100 ng/mL, hGastrin I 0.01 µmol/L, N-acetylcysteine 1.25 mmol/L, nicotinamide 10 mmol/L, PGE2 1 µmol/L, B27 supplement 1 × final, R-spondin1 conditioned media 10% final, and afamin/Wnt3A conditioned media 50% final.

### Cell death assays

For the efficacy studies of the drug candidates, all pancreatic and ovarian cancer cell lines were plated into 96 well plates (1.0 × 10^4^ cells/well) on the day prior to treatment. The cells were treated with escalating doses of either SW43 alone, IAPinh alone, combination of SW43 and IAPinh, or S2/IAPinh. After 24 h of treatment, cell viability was determined using the CellTiter-Glo Luminescent Viability Assay (Promega, Madison, WI). Luminescence signals were recorded using a Multi-Detection Microplate Reader (Synergy HT, BioTek instruments, Winooski, VT). GraphPad Prism 9.0 was used to calculate the half-maximum killing concentrations (IC_50_).

For the studies blocking caspase activities using Z-VAD-FMK (ALX-260-020-M001, Enzo Life Sciences, Farmingdale, NY), a pan-caspase inhibitor, Nuclight Red cell lines (AsPC-1 and OVCAR8) were plated into 96 well plates (1.0 × 10^4^ cells/well) on the day prior to treatment. The cells were treated with or without Z-VAD-FMK (20 µM) 2 h prior to S2/IAPinh (10 µM for AsPC-1 and 8 µM for OVCAR8) treatment. The cells were cultured in phenol red-free medium with 10% FBS, 2 mmol/L of glutamine, and 50 nmol/L of YOYO-1 iodide (Y3601, ThermoFisher Scientific, Kalamazoo, MI) cell death marker during assays. Cell death was measured via the YOYO-1 iodide probe and live cell analysis was measured using Nuclight Red. Cell death was analyzed using IncuCyte FLR imaging system software (Sartorius Ann Arbor, MI). Scalable time-lapse analysis of cell death kinetics (STACK) was used for quantification of cell death, and the lethal fraction was calculated as previously described^54^.

The efficacy study of S2/IAPinh using pancreatic cancer patient-derived organoids was conducted as previously described^44^. Briefly, five hundred viable cells were plated per well in 20 μL 10% Matrigel/ human complete organoid media (n ≥ 3 per cell line). Escalating doses of S2/IAPinh was added 24 h after plating, after the reformation of organoids was visually verified. After 5 days of treatment, cell viability was determined using the CellTiter-Glo Luminescent Viability Assay (Promega, Madison, WI) on a SpectraMax I3 (Molecular Devices, San Jose, CA) plate reader. GraphPad Prism 9.0 was used to calculate the half-maximum killing concentrations (IC_50_).

### Immunohistochemistry (TUNEL and Ki67)

HPAC and OVCAR8 cell lines were treated with SW43 (6 µM), IAPinh (6 µM), S2/IAPinh (6 µM), or vehicle for 6 h. Staining was performed using the Click-iT Plus TUNEL Assay (C10618, Invitrogen, Life Technologies, Eugene, OR) according to the manufacturer’s instructions. F-actin was labeled using ActinGreen 488 ReadyProbes Reagent (R37110, Invitrogen, Life Technologies, Eugene, OR), and DNA using Hoechst (33258, Invitrogen, Life Technologies, Eugene, OR) according to the manufacturer’s instructions. For Ki67 staining, mouse tissue sections (5 μm) were deparaffinized in xylenes, rehydrated in isopropanol, and boiled in antigen unmasking solution (H-3300-350, Vector Laboratories, Newark, CA). After washing with water, the tissues were blocked with 1% bovine serum albumin (A7030, Millipore Sigma, St. Louis, MO), 10% normal donkey serum (D9663, Millipore Sigma, St. Louis, MO) for an hour at room temperature. Tissue specimens were stained overnight at 4 °C with a rabbit anti-Ki67 (ab15580, Abcam, Waltham, MA) at 1:200 dilution. Following washing in Tris Buffered Saline supplemented with 1% bovine serum albumin and 0.1% Tween-20, the tissue sections were incubated for an hour at room temperature with Alexa Fluor 488 donkey anti-rabbit (A21206, Life Technologies, Eugene, OR) at 1:200 dilution. Nuclear staining was performed using Hoechst 33342 (Life Technologies, Eugene, OR) for 5 min, and slides were mounted with Prolong Gold (P36934, Thermo Fisher, Waltham, MA). Fluorescence images were acquired using fluorescent microscope (BX61, Olympus, Bartlett, TN).

### Immunoblotting

HPAC and OVCAR8 cell lines were plated into 6 well plates (3.0 × 10^5^ cells/well) on the day prior to treatment. After the respective treatment, medium was removed and cells were washed two times with PBS. Cells were collected and lysed in RIPA buffer (89900, ThermoFisher Scientific, Kalamazoo, MI) with Halt Protease and Phosphatase Inhibitor Single-Use Cocktail (78443, ThermoFisher Scientific, Kalamazoo, MI) according to the manufacturer’s instructions. Protein concentration of lysates were determined using Pierce BCA Protein Assay Kit (23225, ThermoFisher Scientific, Kalamazoo, MI). Protein expressions of lysates were analyzed using a ProteinSimple Wes instrument (ProteinSimple, Bio-Techne, Minneapolis, MN), according to manufacturer’s protocol. Primary antibodies and dilutions used for the analyses were as follows: cIAP-1 1:2000 for human samples (MAB818, Bio-Techne, Minneapolis, MN), cIAP-1 1:100 for mouse samples (PA5-87514, Invitrogen, Life Technologies, Eugene, OR), cIAP-2 1:500 (NBP-27972, Novus Biologicals, Centennial, CO), X-linked inhibitor of apoptosis protein (XIAP) 1:1000 (610762, BD Biosciences, San Jose, CA), Caspase 3 1:500 (19677–1-AP, Proteintech, Rosemont, IL), Caspase 8 1:50 (NB100-56527, Novus Biologicals, Centennial, CO), and Caspase 9 1:300 (10,380–1-AP, Proteintech, Rosemont, IL). Protein levels were quantified using Compass for SimpleWestern software and normalized by total protein level. All analyses were conducted at least two times.

### Caspase 3/7 activation assay

Nuclight Red cell lines (AsPC-1 and OVCAR8) were plated into 96 well plates (1.0 × 10^4^ cells/well) on the day prior to treatment. The cells were cultured in phenol red-free medium with 10% FBS, 2 mmol/L of glutamine, and 5 µmol/L of IncuCyte Caspase 3/7 Green Dye (4440, Sartorius, Ann Arbor, MI) and treated with either vehicle, SW43 alone, IAPinh alone, combination of SW43 and IAPinh, or S2/IAPinh. Caspase 3/7 activated cells were measured via the IncuCyte Caspase 3/7 Green Dye probe and live cell analysis was measured using Nuclight Red. Cell death was analyzed using IncuCyte FLR imaging system software (Sartorius Ann Arbor, MI). The fraction of caspase 3/7 activated cells was calculated using the following equation: (Caspase 3/7 Green positive cells)/(Caspase 3/7 Green positive cells + NucLight Red positive cells).

### Preclinical mouse models of cancer

We performed murine xenograft experiments according to the animal protocol approved by the Institutional Animal Care and Use Committee (IACUC) at Washington University in St. Louis (protocol No. 20190111). Mice were maintained according to the IACUC guidelines and were anesthetized using isoflurane for all procedures. Six-week-old female C57BL/6 mice, purchased from Charles River (Wilmington, MA) were injected subcutaneously into the right flank with 2 × 10^6^ KP2 cells in 50 µL of serum-free DMEM and combined with 50 µL of Matrigel (356234, Corning, Corning, NY). Six-week-old female athymic nude mice (homozygous for *Foxn1*^*nu*^), purchased from Charles River (Wilmington, MA) were injected subcutaneously into the right flank with 4 × 10^6^ OVCAR8 cells in 50 µL of serum-free RPMI and combined with 50 µL of Matrigel (356234, Corning, Corning, NY). When the tumor reached approximately 100 mm^3^, mice were randomly assigned into five groups (12 mice per group)—1. vehicle (H_2_O/cremophor/DMSO), 2. SW43 alone, 3. IAPinh alone, 4. combination of SW43 and IAPinh, and 5. S2/IAPinh. All treatments were administered daily via the intraperitoneal route (IP). Length and width of the tumors were measured daily with a digital caliper and tumor volumes were calculated using the formula of Tumor Volume = Length × Width^2^ × 0.5. For survival analyses, treatment endpoints were defined as death, tumor volume (1500 mm^3^ for KP2 and 1200 mm^3^ for OVCAR8), or tumor ulceration. All mice were euthanized when tumors grew > 3000 mm^3^ or started ulcerating.

For the assessment of in vivo pathway activation following the various drug treatments, immunoblotting and immunohistochemical staining on tumor tissues were obtained under same conditions that were used as described above for the survival experiment employing the syngeneic KP2 model with six-week-old female C57BL/6 mice. Tumors were surgically removed from euthanized mice 48 h post-treatment, cut into pieces and processed for the respective downstream application.

To evaluate putative toxic side effects of S2/IAPinh treatment, six-week-old naïve C57BL/6 mice (Charles River, Wilmington, MA), were assigned to two groups (total of 6 mice with 3 males and 3 females per group) and treated with either vehicle or S2/IAPinh for a total of 7days (IP route). After this treatment interval, the mice were submitted for necropsy to the Washington University Department of Comparative Medicine. Blood samples were collected for complete blood counts (CBCs) and serum chemistries. Organs were examined grossly and histologically.

### Statistical analyses

All data are presented as the mean values ± SEM and Student’s t test or two-way ANOVA were used to compare variance across groups for statistical significance. Kaplan–Meier analysis was used to draw survival curves with differences tested between groups by the log-rank test. Statistical analyses were performed using SPSS version 13.0 (SPSS, Chicago, IL, USA). *P* < 0.05 was considered to indicate a statistically significant result.

### Ethical approval and consent to participate

All methods were carried out in accordance to the ethics standards of Washington University and are reported in accordance with ARRIVE guidelines (https://arriveguidelines.org). Procedures involving mice were approved by the Washington University Animal Studies Committee (IACUC) (protocol No. 20190111) and conducted in accordance with the guidelines for the care and use of laboratory research animals established by the NIH. For organoid generation, autopsy specimens from metastatic sites were obtained from the Rapid Autopsy Program at University of Nebraska Medical Center and Washington University St. Louis. All tissue donations and experiments were reviewed and approved by the Institutional Review Board of Cold Spring Harbor Laboratory and all clinical institutions. Written informed consent was obtained prior to acquisition of tissue from all patients. The studies were conducted in accordance with recognized ethical guidelines (Declaration of Helsinki).

### Supplementary Information

Supplementary Legends.

Supplementary Figure S1.

Supplementary Figure S2.

Supplementary Figure S3.

Supplementary Figure S4.

Supplementary Figure S5.

Supplementary Table S1.

Supplementary Table S2.

## Data Availability

All data reported in this manuscript are available from the corresponding authors upon reasonable request.
