# Supplementary material for: The novel drug candidate S2/IAPinh improves survival in models of pancreatic and ovarian cancer
Source: Sci Rep. 2024 Mar 16;14:6373. doi: 10.1038/s41598-024-56928-z (PMC10944456; doi:10.1038/s41598-024-56928-z)
Supplement: Supplementary file 1 — Supplementary Legends. [file 41598_2024_56928_MOESM1_ESM.docx]

**The novel drug candidate S2/IAPinh improves survival in models of pancreatic and ovarian cancer.**

**Authors**

Takaomi Hagi^1^, Suwanna Vangveravong^1^, Rony Takchi^1^, Qingqing Gong^1^, S. Peter Goedegebuure^1,2^, Herve Tiriac^3^, Brian A. Van Tine^4^, Matthew A. Powell^2,5^, William G. Hawkins^1,2,*^ and Dirk Spitzer^1,2,*^

**Affiliations**

^1^ Department of Surgery, Washington University School of Medicine, St. Louis, MO, USA

^2^ Alvin J. Siteman Cancer Center, Barnes-Jewish Hospital, and Washington University School of Medicine, St. Louis, MO, USA

^3^ Cold Spring Harbor Laboratory, New York, NY, USA

^4^ Division of Medical Oncology, Washington University School of Medicine, St. Louis, MO, USA

^5^Division of Gynecologic Oncology, Department of Obstetrics and Gynecology, Washington University School of Medicine, St. Louis, MO, USA

**^*^Co-corresponding authors**

Address correspondence to Dirk Spitzer, PhD, Department of Surgery, Washington University School of Medicine, S. Euclid Avenue, St. Louis, MO, USA 63110

Phone: +1 (314) 362 8631, Email: [dmspitzer@wustl.edu](mailto:dmspitzer@wustl.edu) or William Hawkins, MD, Department of Surgery, Washington University School of Medicine, S. Euclid Avenue, St. Louis, MO, USA 63110, Phone: +1 (314) 362 7046, Email: [hawkinsw@wustl.edu](mailto:hawkinsw@wustl.edu)

**Supplementary Figures, Tables and** **Image data to capillary Western blots (WES)**

**Supplementary Table S1** IC_50_ (µM) for each cell lines treated with either SW43, IAPinh, the combination of SW43 plus IAPinh, and S2/IAPinh for 24 hours. Data are shown as means ± SEM, n = 3.

**Supplementary Figure S1** (A) Representative images of TUNEL labeled apoptosis cells in OVCAR8 cells treated with vehicle, SW43 (6 µM), IAPinh (6 µM), or S2/IAPinh (6 µM) for 6 hours. Nuclei were stained in blue with Hoechst, TUNEL positive cells are in red. Scale bars are equal to 50 µm. (B) Quantification of TUNEL positive cells per area in each treatment group. Data are shown as means ± SEM; ****P* < 0.001.

**Supplementary Table S2** (A) Patient characteristics for each patient-derived organoid. (B) IC_50_ (µM) for each organoids treated with S2/IAPinh for 5 days. Data are shown as means ± SEM, n = 4. EUS, endoscopic ultrasound; FNA, fine-needle aspiration; RAP, rapid autopsy.

**Supplementary Figure S2** (A) and (B) Protein expression of cIAP-1, cIAP-2, and XIAP in OVCAR8 cells treated with vehicle, SW43 (10 µM), IAPinh (10 µM), or S2/IAPinh (10 µM) for 6 hours. The precursor and cleaved forms of caspase 3, 8, and 9 were also analyzed for these cells using Wes automated capillary blotting system (Protein Simple). (B) Quantification of protein expression. Relative densitometry of each band normalized to the total protein. Data shown as means ± SEM. ***P* < 0.01, **** *P* < 0.0001. (C) Ratio of Caspase 3/7 counts to NucRed counts in OVCAR8 cells treated with vehicle, SW43 (8 µM), IAPinh (8 µM), combination of SW43 (8 µM) and IAPinh (8 µM), or S2/IAPinh (8 µM) measured by the IncuCyte system (Sartorius). Bar graph shows the ratio of Caspase 3/7 at 48 hours for each treatment. Data shown as means ± SEM. *****P* < 0.001, **P* < 0.05. (D) and (E) Activity of cell death in OVCAR8 cells was measured using YOYO-1 iodide on the IncuCyte (Sartorius). (D) Representative images of OVCAR8 cells treated with or without Z-VAD-FMK and S2/IAPinh (10uM) at baseline and 72 hours after treatment. Scale bars are equal to 20 µm. (E) The AUC of lethal fraction at 72 hours. Data shown as means ± SEM. *****P* < 0.0001.

**Supplementary Figure S3** (A) Complete blood counts and (B) Serum chemistries of six-week-old C57BL/6 mice treated with either vehicle or 25 µmoles/kg/day of S2/IAPinh for 7days. Data are shown as means ± SEM, n ≥ 4. BUN, blood urea nitrogen; AST, aspartate transferase; ALT, alanine transaminase. (C) Summary of histopathological findings of major organs (liver, kidney, lung, and brain) harvested from six-week-old C57BL/6 mice (total of 6 mice with 3 males and 3 females per group) treated with vehicle or 25 µmoles/kg/day of S2/IAPinh for 7days.

**Supplementary Figure S4** OVCAR8 xenograft subcutaneous tumor model using 6-week-old female athymic nude mice (12 mice per group) were start treated when the tumor reached approximately 100 mm^3^. Drug were administered by intraperitoneal injection every day for 35 days with either vehicle (25% cremophor in H_2_O), 30 µmoles/kg/day of SW43 alone, 30 µmoles/kg/day of IAPinh alone, the combination of 30 µmoles/kg/day of SW43 plus 30 µmoles/kg/day of IAPinh, or 30 µmoles/kg/day of S2/IAPinh. (A) Tumor volume of mice during or after each treatment. There were no significant differences between vehicle, SW43, IAPinh, and the combination of SW43 plus IAPinh. Data shown as means ± SEM. ** *P* < 0.01. (C) Kaplan-Meier survival curve of the mice in each treatment group is shown. There were no significant differences in survival between vehicle, SW43, IAPinh, and the combination of SW43 plus IAPinh treated group. ***P* < 0.01. (C) Body weight changes of athymic nude mice during each treatment. There were no significant differences between each treatment group. Data shown as means ± SEM.

**Supplementary Figure S5** (A) Body weight changes of C57BL/6 mice during each treatment: vehicle (25% cremophor in H_2_O); 30 µmoles/kg/day of SW43 alone; 30 µmoles/kg/day of IAPinh alone; the combination of 30 µmoles/kg/day of SW43 and 30 µmoles/kg/day of IAPinh; or 30 µmoles/kg/day of S2/IAPinh. There were no significant differences between each treatment group. Data shown as means ± SEM. (B) Representative images of Ki-67 staining for tumor samples collected 48 hours after the each treatment.

**Image data to capillary Western blots (WES)**

**Figure 4B (WES)**

**Figure 6C (WES)**

**Supplementary Figure S2A (WES)**
