# Supplementary figures and images for: The novel drug candidate S2/IAPinh improves survival in models of pancreatic and ovarian cancer
Source: Sci Rep. 2024 Mar 16;14:6373. doi: 10.1038/s41598-024-56928-z (PMC10944456; doi:10.1038/s41598-024-56928-z)

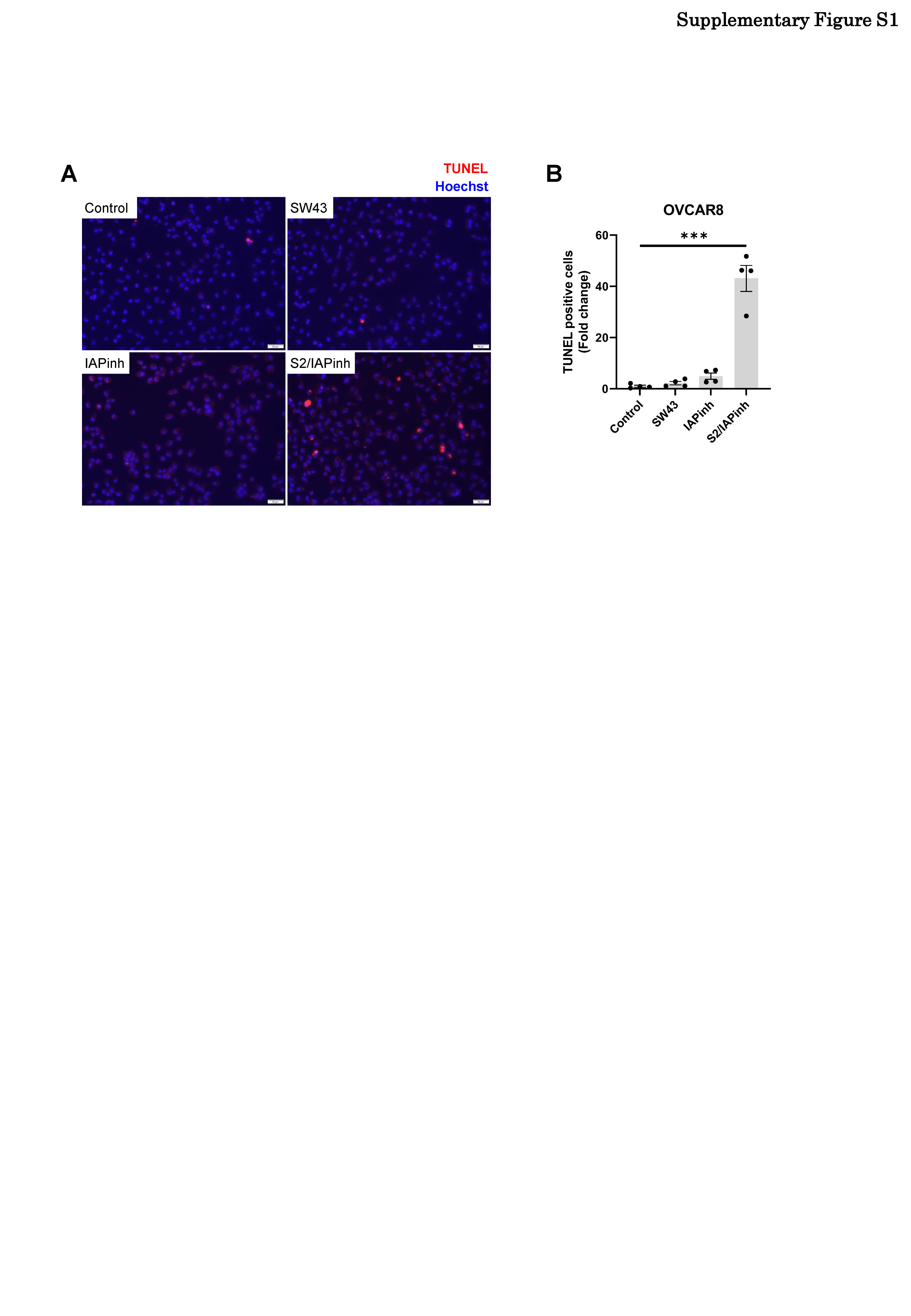

Supplement: Supplementary file 2 — Supplementary Figure S1. [file 41598_2024_56928_MOESM2_ESM.tif]

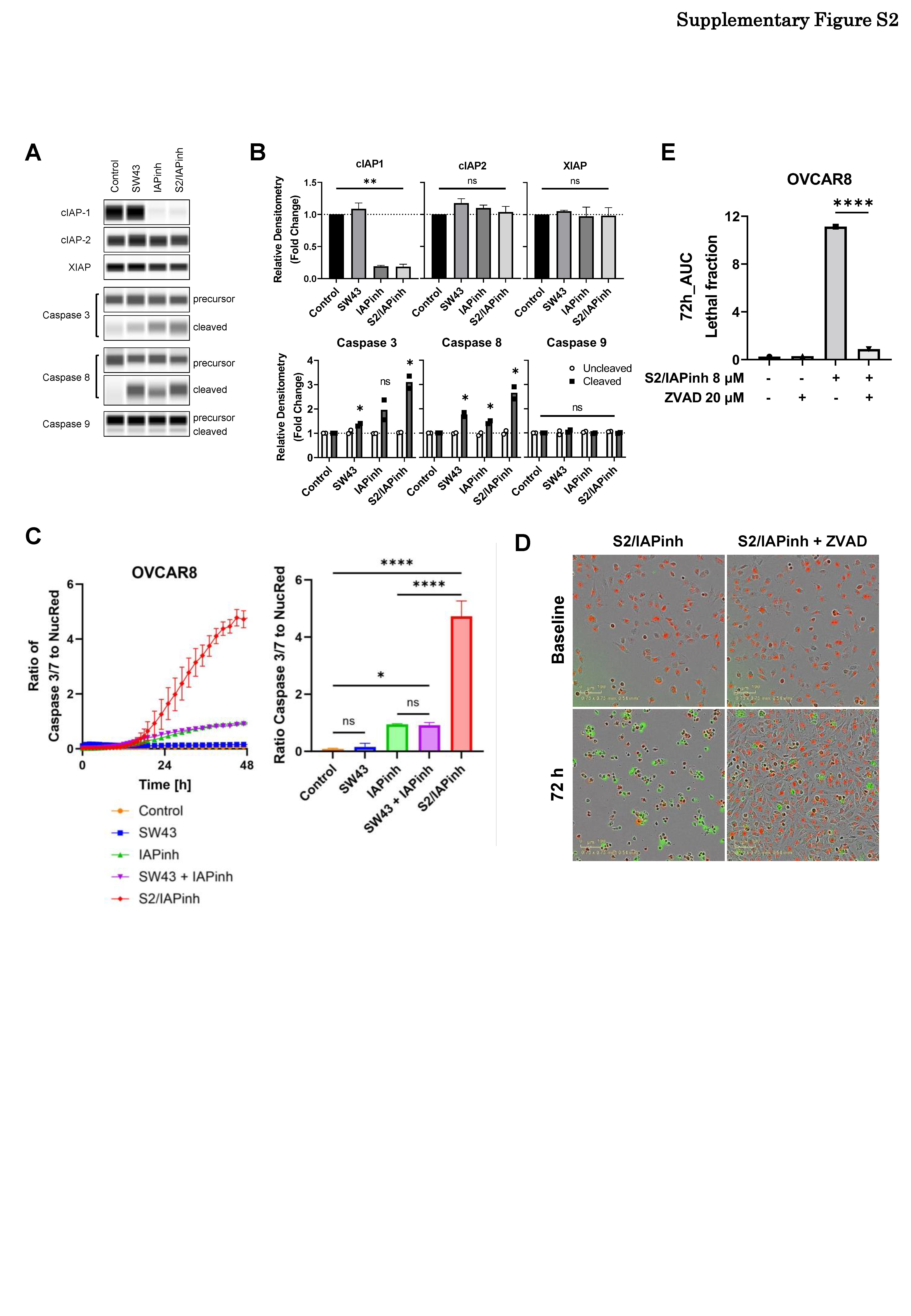

Supplement: Supplementary file 3 — Supplementary Figure S2. [file 41598_2024_56928_MOESM3_ESM.tif]

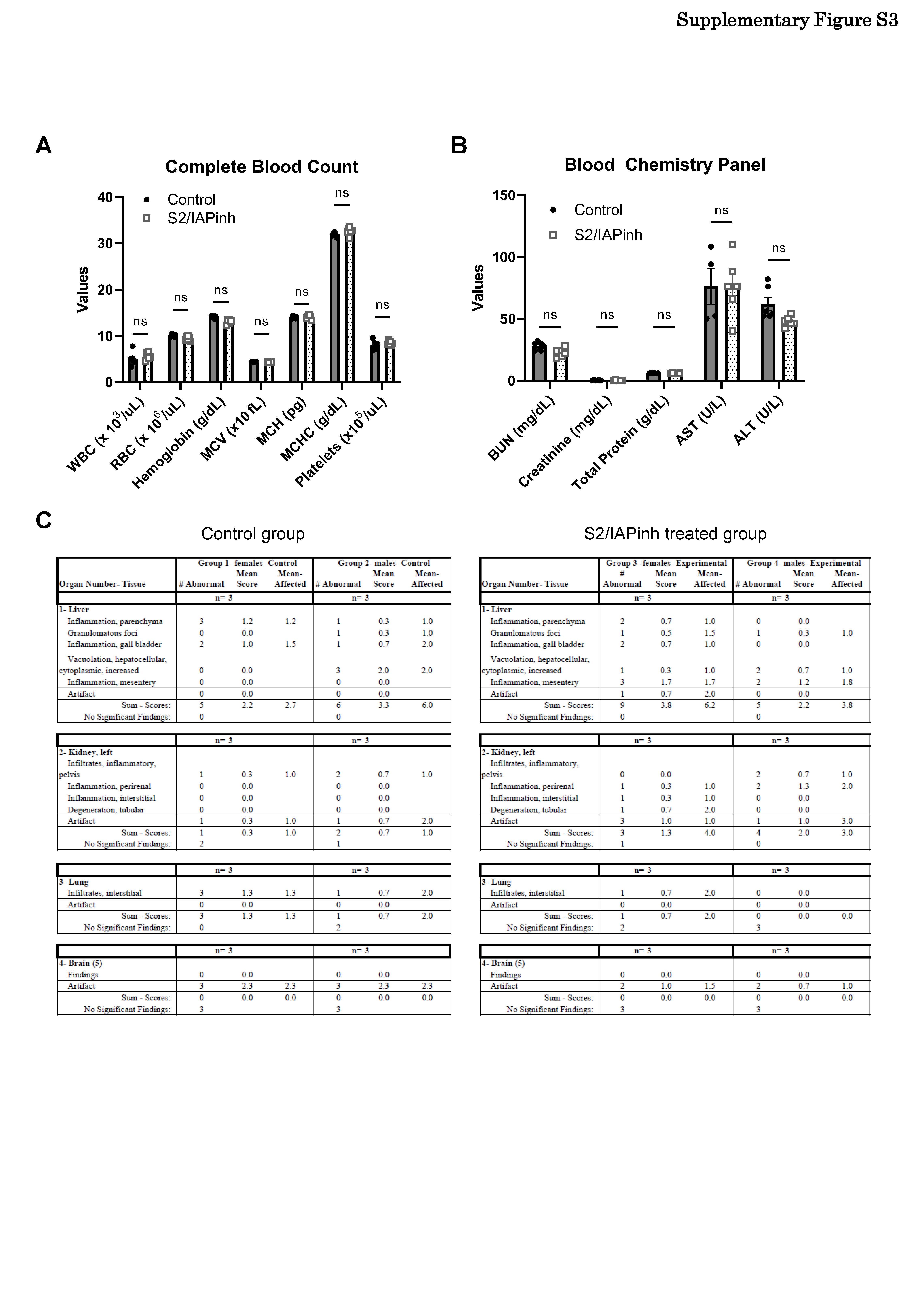

Supplement: Supplementary file 4 — Supplementary Figure S3. [file 41598_2024_56928_MOESM4_ESM.tif]

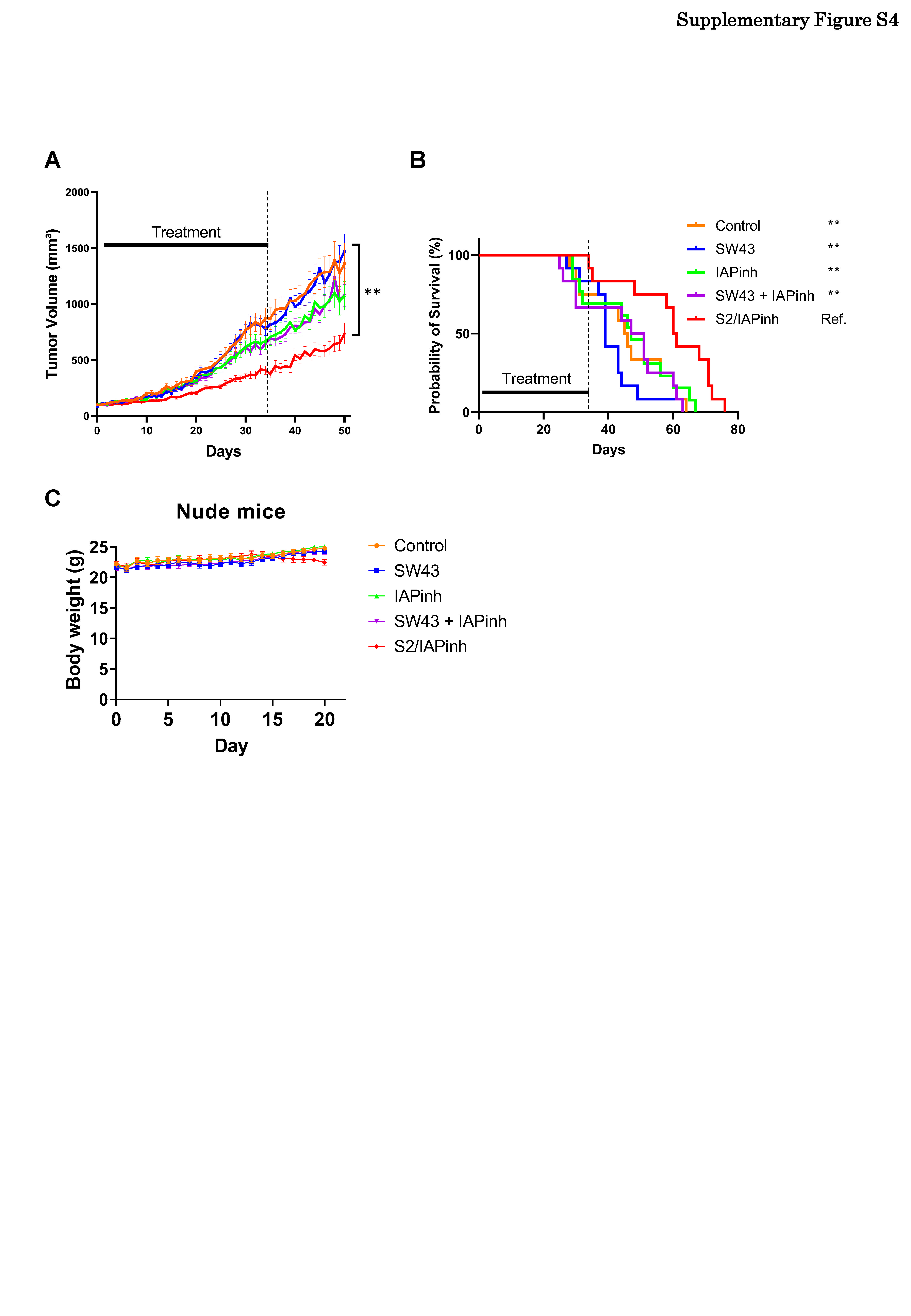

Supplement: Supplementary file 5 — Supplementary Figure S4. [file 41598_2024_56928_MOESM5_ESM.tif]

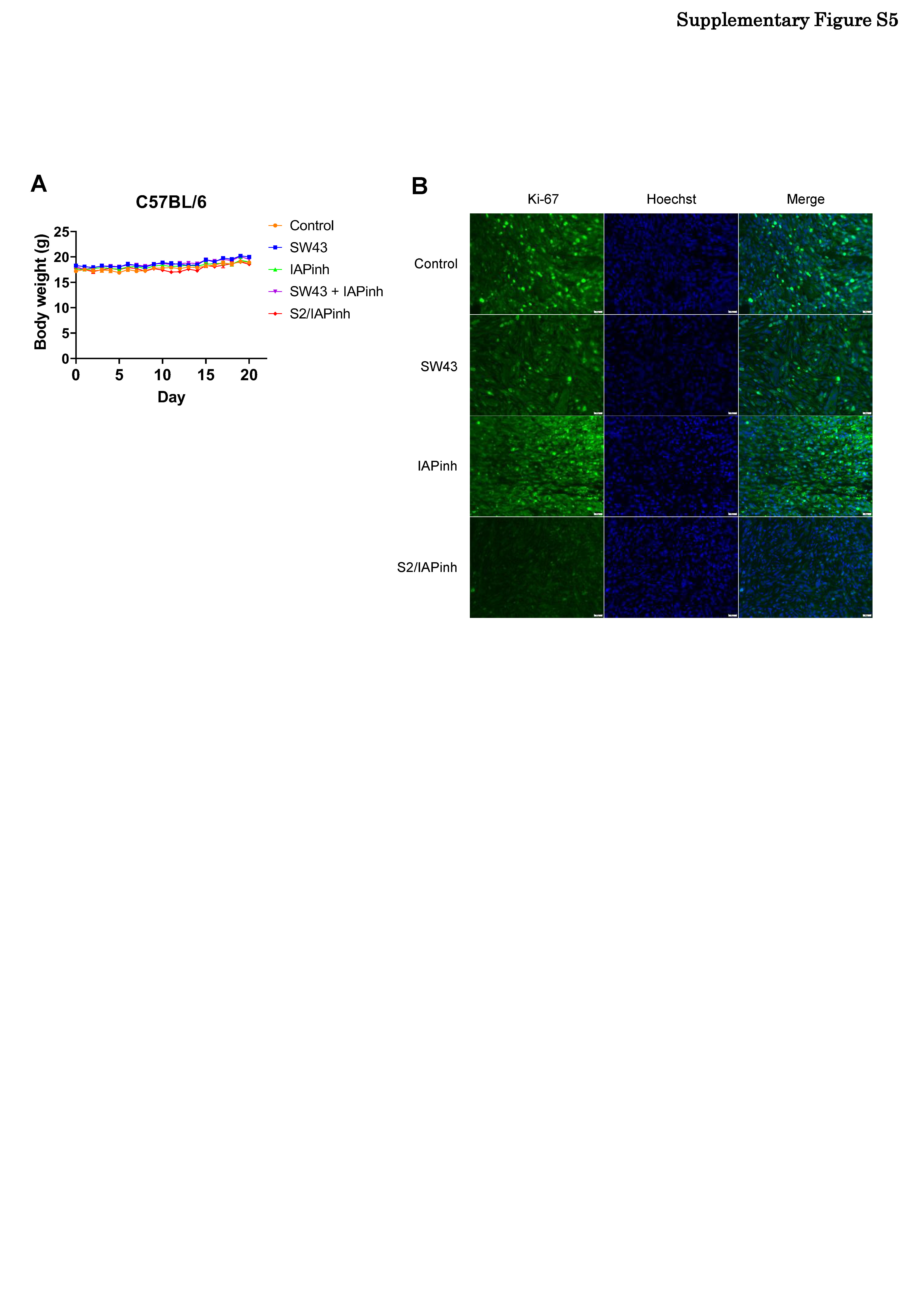

Supplement: Supplementary file 6 — Supplementary Figure S5. [file 41598_2024_56928_MOESM6_ESM.tif]

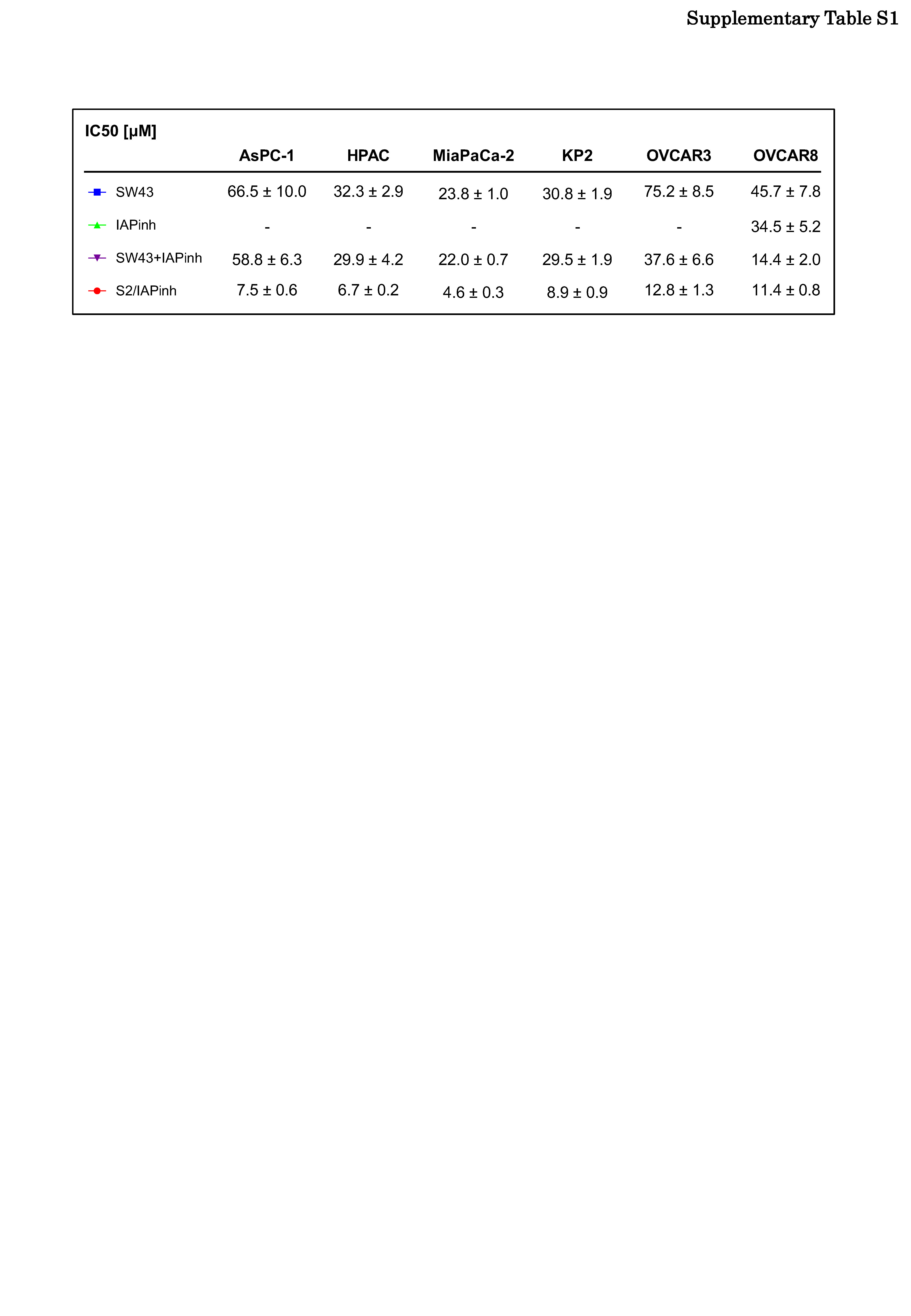

Supplement: Supplementary file 7 — Supplementary Table S1. [file 41598_2024_56928_MOESM7_ESM.tif]

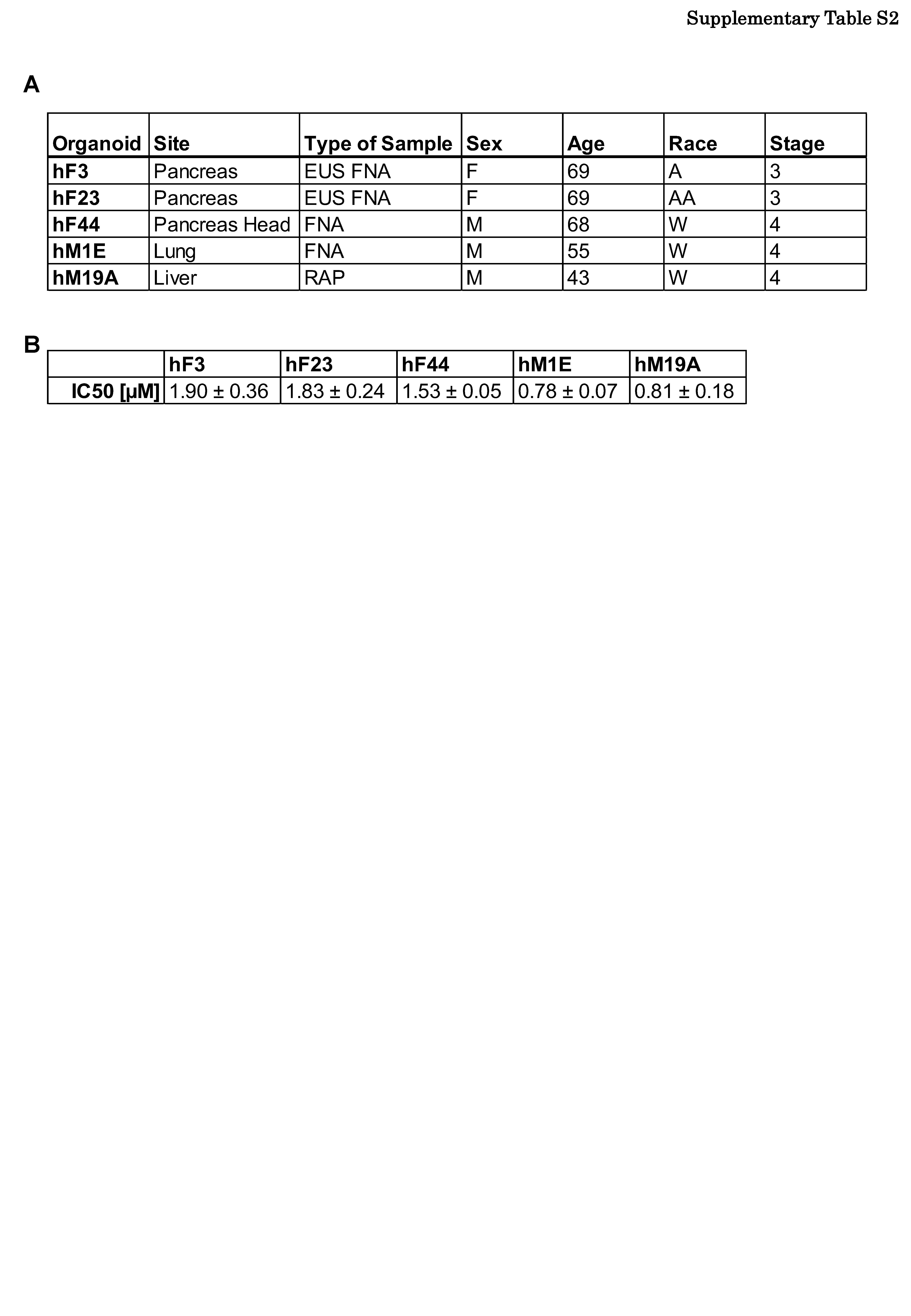

Supplement: Supplementary file 8 — Supplementary Table S2. [file 41598_2024_56928_MOESM8_ESM.tif]
